# Elevated Plasma Tryptophan in Patients with Anorexia Nervosa Compared with Hypotrophic Controls

**DOI:** 10.31662/jmaj.2022-0217

**Published:** 2023-11-27

**Authors:** Hirotaka Shibuya, Takaaki Maruhashi, Yutaro Kurihara, Kento Nakatani, Hisatoshi Arai, Yasushi Asari

**Affiliations:** 1 Department of Emergency and Critical Care Medicine, Kitasato University School of Medicine, Sagamihara, Japan; 2 Department of Psychiatry, Faculty of Medicine, Saitama Medical University, Iruma, Japan

**Keywords:** anorexia nervosa, plasma amino acids, tryptophan metabolism

## Abstract

**Introduction:**

We hypothesized that anorexia nervosa (AN) is associated with pathological amino acid metabolism. This study aimed to identify amino acids exhibiting abnormal metabolism in patients with AN compared with those in low-nutrient controls.

**Methods:**

This was a single-center, retrospective, observational study that compared patients with AN with a low-nutrient control group. All participants were admitted to the Kitasato University Hospital Emergency Center between January 1, 2018, and January 31, 2021. Both the AN and low-nutrient control groups had five patients each. Plasma amino acid category testing was conducted at the same institution for both groups. Patient sex, age, height, weight, and comorbidities were retrospectively extracted. Plasma amino acid fractions, total amino acids, total essential amino acids, total nonessential amino acids, branched-chain amino acids (sum of valine, isoleucine, and leucine), and amino acid concentrations and ratios were compared between the two groups. Data were analyzed using the Mann-Whitney U test.

**Results:**

Body mass index was lower in the AN group (p = 0.00794). Tryptophan levels were significantly higher in the AN group (p = 0.00794). Other amino acid values, the sum of amino acid values, and amino acid ratios were not significantly different between both groups.

**Conclusions:**

Serum tryptophan levels were higher in the AN group than in the low-nutrient group, and AN may be associated with abnormal amino acid metabolism.

## Introduction

Anorexia nervosa (AN) is a severe psychiatric disorder characterized by intentional starvation behavior that leads to malnutrition ^(1)^. AN has one of the highest mortality rates among psychiatric disorders, with more than 50% of lethal cases being associated with clinical and somatic complications ^(2)^. There are two subtypes of AN: restricting (AN-R) and binge/purging (AN-BP) ^(1)^. These subtypes were shown to intermingle and merge, as up to 62% of patients with AN-R also developed AN-BP ^(3)^. Physiological mechanisms of AN subtype integration remain largely unclear, although psychiatric symptoms of AN have been thoroughly investigated ^(4), (5), (6), (7), (8), (9), (10)^.

Changes in brain neurotransmitter signaling, including modulation of serotonin and dopamine levels, have been found to be associated with the psychiatric symptoms of AN ^(7)^. Low serotonin levels have been implicated in the pathomechanism of several mental deficiencies, including depression, deficits in motivation, and psychosis ^(7), (11), (12)^. These mental complications are highly prevalent in patients with AN ^(7), (11), (12)^. Additionally, other AN-linked behavioral characteristics (behavioral dysregulation), including hyperactivity, body image and weight delusions, and excessive exercise, have been attributed to the overactivity of the dopamine system ^(1), (7)^. Notably, serotonin inhibits the dopamine system via the activation of gamma-aminobutyric acid signaling ^(7), (11)^.

The precursors of serotonin and dopamine are tryptophan and tyrosine, respectively ^(13), (14)^. Tyrosine is synthesized from phenylalanine, an essential amino acid ^(4)^. Essential amino acid deficiency is associated with starvation in patients with AN and is thought to cause psychiatric symptoms ^(4), (5), (6), (7), (8)^. However, previous studies analyzing amino acids in patients with AN found no significant differences in the total amount of essential amino acids between the AN and healthy control groups ^(9), (10)^. The factors that protect patients with AN from having a reduced total amount of essential amino acids, despite inadequate dietary intake, have not yet been elucidated.

Amino acid levels are elevated in the presence of abnormalities in amino acid metabolism ^(15)^. We hypothesized that patients with AN have abnormal amino acid metabolism that helps maintain sufficient levels of total essential amino acids despite dietary deficiencies. However, the amino acid metabolism in patients with AN remains to be investigated. Several previous studies have measured and compared the amino acid composition between AN and healthy control groups ^(5), (6), (8)^. Therefore, in our study, we aimed to compare the AN group with a low-nutrient group of patients (control group) diagnosed with diseases other than AN. The choice of the low-nutrient control group allows elimination of the element of inadequate dietary intake and the identification of amino acids exhibiting abnormal metabolism in the AN group. The present study identified differences in amino acid composition between patients with AN and controls on a low-nutrient diet.

## Materials and Methods

### Study design and ethical consideration

This was a single-center retrospective observational study. Data from the study participants were extracted from electronic medical records. Information on height, weight history, body mass index (BMI), medical history, and blood test results were obtained. The Ethics Committee of Kitasato University School of Medicine and Hospital approved this study (Approval Number: B20-217). This committee waived the need for informed consent because of its retrospective design.

### Patient selection and characteristics

The selection criteria for patients with AN were as follows:1) admission to the Emergency and Disaster Medical Center of Kitasato University Hospital between January 1, 2018, and January 31, 2021, and 2) diagnosis of AN. Patients were diagnosed by a psychiatrist according to the criteria indicated in the American Psychiatric Association Diagnostic and Statistical Manual of Mental Disorder (4th Edition DSM-IV) ^(1)^. The exclusion criteria were as follows:1) age ≤ 15 years, 2) inborn errors of metabolism, and 3) patients who had received nutritional supplementation before admission. The selection criteria for the control group were as follows:1) patients who had an amino acid fractionation test performed at this institution between January 1, 2018, and January 31, 2021; 2) BMI < 18 m^2^/kg, or serum albumin < 3.4 mg/dl ^(16)^, and 3) females; of note, patients with AN were mostly females ^(13), (14)^. The exclusion criteria for the control group were as follows:1) age ≤ 15 years, 2) inborn errors of metabolism, 3) eating disorders, and 4) a diagnosis of a central nervous system disease.

### Specimen collection and analyses

Blood samples were obtained from all patients after an overnight fast and stored in test tubes sealed with ethylenediaminetetraacetic acid. On the day of blood collection, the amino acid fractionation test was outsourced to SRL Corporation (Tokyo, Japan). The results for each amino acid category were available within two weeks.

### Statistical analysis

Continuous variables, including patient background, amino acid levels, and amino acid ratios, were subjected to the Mann-Whitney U test, a nonparametric t-test, with the level of statistical significance set at p < 0.05. EZR statistical analysis software (Saitama Medical Center, Jichi Medical University, Saitama, Japan) was used for the analysis. Total amino acids, total essential amino acids, total nonessential amino acids, branched-chain amino acids (BCAAs; sum of valine, isoleucine, and leucine), nonessential amino acids/essential amino acids ratio, phenylalanine/tyrosine ratio (an indicator of muscle catabolism ^(17)^), glycine/valine ratio (an indicator of low nutrition ^(18)^), glycine/BCAA ratio (an indicator of protein intake ^(19)^), alanine/BCAA ratio (an indicator of energy intake ^(19)^), alanine/valine ratio (an indicator of energy intake ^(19)^), methionine/cystine ratio (an indicator of sulfur metabolism ^(20)^), and tryptophan/LNAA (LNAA: sum of the large neutral amino tyrosine, phenylalanine, leucine, isoleucine, and valine) ratio (an indicator of brain transfer of tryptophan ^(7)^) were calculated.

## Results

During the study period, five patients were identified to be suitable according to the selection criteria for the AN group. All patients were diagnosed with an acute AN-R type of illness. The control group also consisted of five patients. The BMI of patients in the AN group was lower (p = 0.00794) than that of patients in the control group (Table 1). The underlying diseases in the control group were psoriatic arthritis, nummular dermatitis, alcoholism, necrolytic migratory erythema, and whole-body bruising. In addition to the diagnostic differences and accompanying diseases, there were no significant differences in patient backgrounds between the groups. Tryptophan levels were significantly higher in the AN group (p = 0.00794). No significant differences were observed between the groups for the other amino acid values (Table 2). There were no significant differences in the total amino acids, total BCAA, total essential amino acids, or nonessential amino acid contents between both groups. Likewise, no significant differences were observed in the ratios of nonessential amino acid/essential amino acids, phenylalanine/tyrosine, glycine/valine, glycine/BCAA, alanine/BCAA, alanine/valine, methionine/cystine, and tryptophan/LNAA (Table 3).

**Table 1.** Backgrounds of the Two Groups.

	AN, median (IQR)	Control, median (IQR)	P-value
Number of patients	5	5
Women%	100%	100%	NS
Age (year)	34 (26-35)	42 (41-42)	NS
BMI (kg/m^2^)	10.8 (10.1-12.5)	17.9 (17.6-22.1)	0.00794
Serum albumin (mg/dL)	3.8 (3.8-3.9)	2.7 (2.6-3.3)	NS

AN: anorexia nervosa, BMI: body mass index, NS: not significant, IQR: interquartile range

**Table 2.** Serum Amino Acids of the Two Groups.

	(μmol/L)	AN, median (IQR)	control, median (IQR)	P-value
EAA	Valine	224.6 (136.2-247.0)	108.4 (98.6-141.3)	NS
	Leucine	139.9 (69.1-149.5)	64.1 (53.2-88.5)	NS
	Isoleucine	65.3 (26.2-69.9)	38.8 (24.3-43.8)	NS
	Phenylalanine	78.4 (61.5-78.8)	68.8 (56.8-72.0)	NS
	Tryptophan	59.4 (47.6-69.9)	39.3 (29.7-44.3)	0.00794
	Threonine	78.3 (57.5-99.2)	83.9 (63.4-116.1)	NS
	Methionine	18.7 (17.9-24.7)	17.9 (16.2-22.6)	NS
	Histidine	54.0 (50.4-56.4)	60.2 (48.5-69.7)	NS
	Lysine	117.8 (99.8-188.5)	121.7 (96.1-185.1)	NS
NEAA	Tyrosine	72.9 (56.6-83.7)	49.6 (24.1-61.9)	NS
	Taurine	35.2 (33.5-51.9)	28.9 (27.3-39.3)	NS
	Serine	46.6 (44.10-74.0)	49.6 (41.7-92.9)	NS
	Asparagine	37.9 (35.3-44.1)	24.5 (17.3-40.6)	NS
	Glutamine	433.2 (388.5-460.7)	498.6 (209.6-580.9)	NS
	Glutamic acid	19.5 (17.0-20.8)	18.4 (11.6-28.8)	NS
	Proline	57.7 (40.8-71.7)	88.3 (59.9-109.4)	NS
	Glycine	140.1 (135.4-170.6)	189.1 (128.1-230.0)	NS
	Alanine	113.3 (111.6-269.2)	276.8 (112.3-279.1)	NS
	Citrulline	12.6 (10.9-14.3)	13.7 (10.9-33.9)	NS
	Cystine	30.5 (28.6-38.2)	11.7 (10.2-48.9)	NS
	Ornithine	29.7 (24.0-35.8)	41.4 (15.1-42.5)	NS
	Arginine	38.2 (37.7-55.5)	85.8 (34.0-86.7)	NS

AN: anorexia nervosa, NS: not significant, IQR: interquartile range, EAA: essential amino acid, NEAA: nonessential amino acid

**Table 3.** Amino Acids Analysis of the Two Groups.

	AN, median (IQR)	control, median (IQR)	P-value
Total AA (μmol/L)	1885.0 (1597.1-1971.8)	2061.7 (1246.2-2544.1)	NS
NEAA (μmol/L)	1038.3 (1038.1-1321.4)	1364.1 (740.1-1659.9)	NS
EAA (μmol/L)	791.9 (664.1-851.3)	698.7 (508.4-699.6)	NS
BCAA (μmol/L)	439.4 (228.2-456.8)	211.3 (176.1-273.6)	NS
LNAA (μmol/L)	549.4 (379.5-461.5)	354.5 (294.5-419.1)	NS
EAA/NEAA	0.58 (0.50-0.82)	0.54 (0.51-0.69)	NS
phenylalanine/tyrosine	1.08 (0.83-1.27)	1.39 (1.16-1.95)	NS
glycine/valine	0.97 (0.62-1.25)	1.34 (1.30-1.38)	NS
glycine/BCAA	0.55 (0.32-0.75)	0.70 (0.69-0.73)	NS
alanine/BCAA	0.31 (0.25-0.99)	0.82 (0.64-1.01)	NS
alanine/valine	0.63 (0.50-1.75)	1.61 (1.14-1.96)	NS
methionine/cystine	0.86 (0.49-1.79)	1.03 (0.46-1.38)	NS
tryptophan/LNAA	0.12 (0.09-0.16)	0.11 (0.10-0.13)	NS

AA: amino acids, NEAA: nonessential amino acids, EAA: essential amino acids, BCAA: branched chain amino acids, LNAA: large neutral amino acids

## Discussion

The main difference between the present study and previous investigations is associated with the comparison approach and choice of control group. In this study, the AN group data were compared with the low-nutrient control group data instead of a healthy control group data. While comparing the patients’ background characteristics, we found that BMI was lower in the AN group, but not all amino acid ratios (indicators of nutritional status) were significantly different between the two groups. The nutritional status of the patients in both groups was comparable in terms of amino acid fractions. Although BMI in the AN group was lower than that in the low-nutrient control group, serum tryptophan concentration was significantly higher in the AN group, which may indicate abnormal amino acid metabolism in AN.

Contrary to our findings, serum tryptophan concentration in patients with AN was previously found to be lower than that in healthy controls ^(21)^. Similar results were observed in previous studies ^(5), (6), (8)^. However, all of these studies compared the data of patients with AN with those of healthy controls. In our study, we compared the serum tryptophan concentration in the AN group with that in a control group of patients who also had low concentrations of nutrients in the blood. Our study found that patients with AN have a higher level of serum tryptophan than the low-nutrient group, suggesting that patients with AN have abnormal tryptophan metabolism.

Ingested tryptophan is metabolized via three protein-related pathways: the kynurenic metabolic pathway, serotonin synthesis, and protein synthesis ^(7)^. Because 90% of tryptophan is metabolized via the kynurenic pathway, it is the most important pathway for tryptophan degradation ^(22)^. The kynurenic pathway metabolism of tryptophan is regulated by the rate-limiting enzyme, indoleamine 2,3-dioxygenase 1 (IDO-1) ^(23)^. IDO-1 activity is inhibited by nitric oxide (NO) ^(24)^. It has been shown that elevated NO inhibits IDO-1 activity and increases serum tryptophan levels. Accordingly, hay fever and asthma increase NO and serum tryptophan concentrations ^(25)^. However, it has not been tested whether patients with any underlying disease and/or low-nutrients have increased NO levels. Notably, elevated NO levels have been reported in AN ^(26)^. The pathogenesis of increased NO production is linked to elevated levels of inflammatory mediators and increased catabolism associated with AN starvation ^(26)^. Increased serum tryptophan levels in the AN group may be caused by IDO-I activity inhibition via increased NO production; however, this hypothesis remains to be tested.

**Figure 1.**
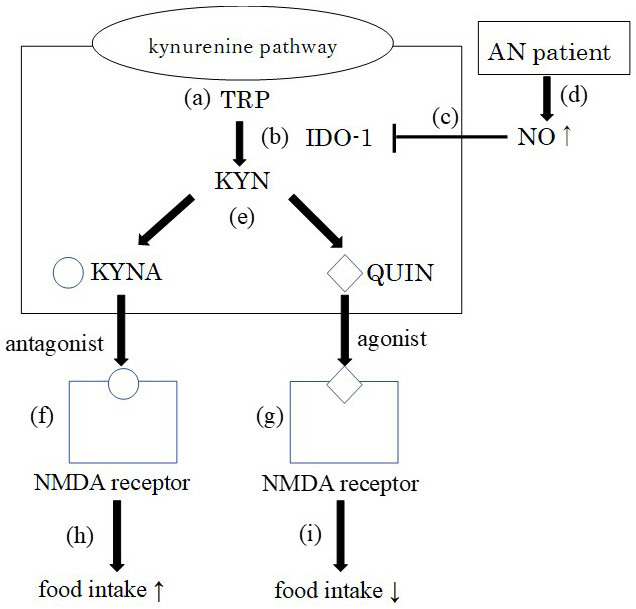
Relationship between the kynurenine pathway, N-methyl-d-aspartate receptor, and food intake. (a) In the kynurenine pathway, tryptophan (TRP) is metabolized into kynurenine (KYN). (b) The kynurenic pathway metabolism of tryptophan is regulated by the rate-limiting enzyme, indoleamine 2,3-dioxygenase 1 (IDO-1). (c) IDO-1 activity is inhibited by nitric oxide (NO). (d) Patients with anorexia nervosa (AN) have elevated NO levels. (e) Kynurenic acid (KYNA) and quinolinic acid (QUIN) are metabolites of kynurenine. (f) KYNA is an antagonist of the N-methyl-d-aspartate (NMDA) receptor. (g) QUIN is an agonist of the NMDA receptor. (h) NMDA agonists decrease food intake. (i) NMDA antagonists increase food intake.

Metabolites of the kynurenine pathway are involved with N-methyl-d-aspartate (NMDA) receptors in the brain. The neurotransmitters quinolinic acid (QUIN) and kynurenic acid (KYNA), which are metabolites of kynurenine, are agonists and antagonists of NMDA receptors, respectively ^(27)^. NMDA receptor stimulation is involved in the activation of satiety centers. Therefore, NMDA agonists decrease food intake ^(28)^, whereas NMDA antagonists increase it ^(29), (30)^. The altered balance between QUIN and KYNA levels during kynurenine metabolism may alter food intake (Figure 1). Although the physiological mechanisms of the merging of AN-R and AN-BP have not been elucidated, the imbalance in QUIN and KYNA levels associated with abnormalities in the kynurenine pathway should be addressed as a potential mechanism of AN subtype transformation.

This study had several limitations. First, the statistical results were questionable because of the limited number of participants in the study. Nonparametric tests with a small number of cases may not produce significant differences. Therefore, we cannot deny the possibility that items that do not show significant differences in this comparison may show significant differences if the number of cases is increased. Second, selection bias was possible due to the retrospective nature of this observational study. Third, the median age of the recruited patients with AN was higher than those reported in previous studies ^(9), (10)^. Interestingly, serum tryptophan concentrations decrease with age ^(31)^. A few other reports have indicated age-related changes in amino acid composition ^(32)^, which may have affected the results. Further studies with younger patients are required to confirm these results. Fourth, dehydration is present in the acute phase of AN, which may have affected amino acid concentrations. Since the AN group in this study had blood test data from the day after the visit, dehydration correction may not have been completed, and the possibility that the AN group’s amino acid concentrations were displayed higher cannot be ruled out. Fifth, the control group were not healthy subjects, but a low-nutrient group with background diseases, including inflammatory diseases, which may have affected the amino acid metabolism of the control group. Sixth, the present retrospective study lacked information on the period of AN and Control group malnutrition and changes after regaining weight. Measuring amino acid values corresponding to changes in the nutritional status of patients with AN over time would contribute to the future elucidation of amino acid metabolism in AN.

### Conclusions

Patients with AN had higher blood tryptophan levels than patients in the low-nutrient group. Based on the differences in tryptophan levels between the preselected groups of patients, we hypothesized that AN is associated with abnormal tryptophan metabolism. Abnormalities in tryptophan metabolism in AN may contribute to our understanding of the pathophysiology of AN.

## Article Information

### Conflicts of Interest

None

### Acknowledgement

Enago (www.enago.jp) checked this article for the English language review.

### Author Contributions

Conception and drafting of the manuscript: HS and TK.

Acquisition of data and data analysis: YK and KN.

Critical revision of the manuscript for important intellectual content: HA and YA.

All authors have approved the final version of the manuscript for publication.

### Approval by Institutional Review Board (IRB)

Approval Number: B20-217

Institution: Kitasato University School of Medicine and Hospital.
